# Association of hsCRP, White Blood Cell Count and Ferritin with Renal Outcome in Chronic Kidney Disease Patients

**DOI:** 10.1371/journal.pone.0052775

**Published:** 2012-12-31

**Authors:** Yi-Chun Tsai, Chi-Chih Hung, Mei-Chuan Kuo, Jer-Chia Tsai, Shih-Meng Yeh, Shang-Jyh Hwang, Yi-Wen Chiu, Hung-Tien Kuo, Jer-Ming Chang, Hung-Chun Chen

**Affiliations:** 1 Division of Nephrology, Kaohsiung Medical University Hospital, Kaohsiung, Taiwan; 2 Faculty of Renal Care, Kaohsiung Medical University, Kaohsiung, Taiwan; 3 Department of Internal Medicine, Kaohsiung Municipal Hsiao-Kang Hospital, Kaohsiung, Taiwan; University of Leicester, United Kingdom

## Abstract

Inflammation is a pathogenic factor in renal injury, but whether inflammation is related to renal outcome in chronic kidney disease (CKD) patients is little known. We thus assess the association of inflammation and renal outcome in an advanced CKD cohort. This study analyzed the association between inflammatory markers, such as C-reactive protein (hsCRP), white blood cell (WBC) count and ferritin, renal replacement therapy (RRT) and rapid renal progression (estimated GFR slope<-6 ml/min/1.73 m^2^/y) in 3303 patients with stage 3–5 CKD. In all subjects, the mean hsCRP, WBC count, and ferritin levels were 1.2 (0.4, 5.4) mg/L, 7.2±2.3×10^3^ cells/µL, and 200 (107,349) ng/mL, respectively. During a mean 3.2-year follow-up, there were 1080 (32.7%) subjects commencing RRT, and 841(25.5%) subjects presenting rapid renal progression. Both hsCRP and ferritin were associated with increased risk for RRT with the adjusted HR (tertile 3 versus tertile 1∶1.17 〔1.01–1.36〕 and 1.20 〔1.03–1.40〕, respectively). Both hsCRP and ferritin were associated with increased odds for rapid renal progression with the adjusted OR (tertile 3 versus tertile 1∶1.40 〔1.13–1.77〕 and 1.32 〔1.06–1.67〕, respectively). hsCRP and ferritin stratified by albumin were also associated with RRT and rapid renal progression. Instead, WBC count was not associated with renal outcome. In conclusion, elevated levels of hsCRP and ferritin are risk factors associated with RRT and rapid renal progression in advanced CKD patients.

## Introduction

Chronic kidney disease (CKD) has been recognized as a worldwide health threat [1] and understanding its complex pathophysiological mechanisms would help greatly in taking care of patients with CKD. Irrespective of the causes, most CKD presentations share common histological presentations of glomerulosclerosis, tubulointerstitial fibrosis and vascular sclerosis [2]. Studies have identified inflammation as an important pathogenic factor in renal injury. Inflammation mediates the processes of monocyte influx, proliferation of macrophages and matrix expansion, and results in glomerulosclerosis and tubulointerstitial injury [3]–[5] which may further exacerbate kidney injuries. Inflammation may stimulate glomerular cells to increase production and reduce degradation of extracellular matrix protein, leading to glomerular hypertension, tubulointerstitial fibrosis and renal scarring [6].

Accumulating clinical evidence has demonstrated that inflammation is one of the major causes of poor outcome in patients with renal failure. For example, the elevated level of C-reactive protein (CRP) is indicative of an inflammatory response and it is now widely accepted as a marker of atherosclerosis. The increase in CRP has been associated with all-cause and cardiovascular mortality in patients on dialysis or not [7]–[10]. Besides, white blood cell (WBC) count is also a traditional indicator of inflammation and infection responses, and previous studies revealed the significant association of WBC count and adverse outcome in dialysis patients [11]. Ferritin, as a clinical marker of iron storage, is often influenced by inflammation. Ferritin induces macrophage accumulation during inflammation and increases reactive oxygen species (ROS) formation [12]. Ferritin is significantly associated with mortality and cardiovascular outcome in patients with renal failure [13].

Previous studies showed inconsistent results for association between inflammation and progression of kidney function in general population [14]–[19]. The result of a significant association between CRP and lower eGFR in non-diabetes in PREVEND study (Prevention of Renal and Vascular ENd stage Disease) [14] was similar to that in African American cohort of the Jackson Heart study [15]. In contrast, Shankar et al. indicated that elevated levels of CRP were not associated with progression to CKD in a population-based cohort [19]. The Atherosclerosis Risks in Communities (ARIC) study showed that increased WBC count was correlated with greater risk for renal function progression [16], but Kovesdy et.al revealed the opposite observation [20]. However, in CKD cohort not on dialysis, the possible relationship of inflammation and the risk for renal function decline or progression to dialysis is little known. Therefore, this study tests the hypothesis whether inflammatory markers, such as high sensitivity CRP (hsCRP), WBC count and ferritin are associated with renal outcome (renal function decline and progression to dialysis) in stages 3–5 CKD patients.

## Materials and Methods

### Study Participants

This retrospective study was conducted at a medical center and a regional hospital in Southern Taiwan. 3749 patients in our integrated CKD program from 11 November 2002 to 31 May 2009 were included and followed until 31 May 2010 [21]. CKD was staged according to K/DOQI definitions and the eGFR was calculated using the equation of the 4-variable Modification of Diet in Renal Disease (MDRD) Study [22]. We excluded patients requiring maintenance dialysis, 90 patients who were lost to follow-up in less than three months and 356 patients in CKD stages 1–2. The final study population comprised 3303 CKD stages 3–5 patients.

### Ethics Statement

The study protocol was approved by the Institutional Review Board of the Kaohsiung Medical University Hospital (KMUH-IRB-990198). Informed consents were obtained in written form from patients and all clinical investigation was conducted according to the principles expressed in the Declaration of Helsinki. The patients gave consent for the publication of the clinical details.

### Data Collection

Baseline demographic and clinical data were obtained from medical records and interviews with patients at enrollment. hsCRP was measured in serum using near infrared particle immunoassay rate method by Beckman Coulter UniCel-DxC 800 (Beckman Coulter). WBC count was determined using electromagnetic impedance method by Beckman Coulter LH 755 (Beckman Coulter). Ferritin was measured in serum using two-site immunoenzymatic assay by Beckman Coulter UniCel-Dxl 800 (Beckman Coulter). Diabetes mellitus (DM) and hypertension were defined as those with a medical history through chart review. Cardiovascular disease (CVD) was defined as a history of heart failure, acute or chronic ischemic heart disease, and cerebrovascular disease. Autoimmune disease was defined as a history of systemic lupus erythematosus, rheumatic arthritis and other collagen vascular disease. The use of statins was defined as patients using statins for more than three months before and after enrollment. The mean arterial pressure (MAP) was calculated by the averaged systolic and diastolic blood pressure measured three months before and after enrollment, using one-third averaged systolic blood pressure plus two-thirds averaged diastolic blood pressure. The laboratory data three months before and after enrollment of the CKD care system were averaged and analyzed.

### Renal Outcome

Two major outcomes were accessed: renal replacement therapy (RRT) and rapid renal progression. The RRT was confirmed by reviewing medical charts or catastrophic illness certificate (issued by the Bureau of National Health Insurance in Taiwan) and defined as requiring maintenance hemodialysis, peritoneal dialysis, or renal transplantation. The timing for RRT was considered according to the regulations of the Bureau of the National Health Insurance of Taiwan regarding the laboratory data, eGFR, uremic status, and nutritional status [23]. The decline in kidney function was assessed by the eGFR slope, defined as the regression coefficient between eGFR and time in units of ml/min per 1.73 m^2^ per year. At least three eGFR values were required to estimate the eGFR slope. All eGFR values available in two hospitals by the end of the observation period were included for calculation. Rapid renal progression was defined as the lowest quartile (the eGFR slope<–6 ml/min/1.73 m^2^ per year, an integer near the cutoff point between the lowest two quartiles of the eGFR slope) [21]. Patients were censored at the commencement of RRT, death, or the end of follow-up.

### Statistical Analysis

Statistical results of baseline characteristics of all subjects were stratified by tertiles of hsCRP, cut at 0.5 and 3 mg/L. The tertile of ferritin was cut at 132 and 288 ng/mL. The tertile of WBC count was divided at 6 and 7.7×10^3^/µL. Continuous variables were expressed as mean±SD or median (25^th^, 75^th^ percentile), as appropriate, and categorical variables were expressed as percentages. Skewed distribution continuous variables were log-transformed to attain normal distribution. The significance of differences in continuous variables among groups of tertiles was tested using one-way ANOVA analysis or Kruskall-Wallis analysis, as appropriate. The difference in the distribution of categorical variables was tested using the Chi-square test. Multivariate linear regression was used to identify the factors associated with log-transformed hsCRP. Cox regression models were utilized to examine the relationship between RRT and tertiles of hsCRP, WBC count and ferritin. Multivariable logistic regression models were also utilized to evaluate the association of rapid renal progression with tertiles of hsCRP, WBC count and ferritin. Besides, sensitivity analysis was conducted to exclude the influence of the wide range of hsCRP tertiles on renal outcome. We reorganized our cohort by hsCRP, divided at 0.5, 3, and 10 mg/L. We also reorganized our cohort by hsCRP, divided at 1 and 3 mg/L based on recommendation from the American Heart Association, for sensitivity analysis [24]. In order to eliminate the influence of the interaction between inflammation and albumin on renal outcome, hsCRP, WBC count and ferritin were stratified by albumin deciles for analysis. The adjusted covariates were hierarchically followed: model (1) age, gender, eGFR, urine protein-creatinine ratio (PCR); model (2) plus glycated hemoglobin, MAP, DM, CVD, current smoking status, statins use; model (3) plus serum hemoglobin, albumin, log-transformed cholesterol, phosphorus, body mass index (BMI); and model (4) plus causes of CKD. Statistical analyses were conducted using SPSS 18.0 for Windows (SPSS Inc., Chicago, Illinois). Statistical significance was set at a two-side p-value of less than 0.05.

## Results

### Characteristics of Entire Cohort

The mean age of study subjects was 63.5±13.5 years and 42.2% were male. The mean levels of hsCRP, WBC count and ferritin were 1.2 (0.4, 5.4) mg/L, 7.2±2.3×10^3^/µL, and 200 (107,349) ng/mL, respectively. Table 1 shows the baseline clinical characteristics organized by tertiles of hsCRP, namely those with hsCRP divided at 0.5 and 3 mg/l. The tertile 3 had the highest proportion of CKD stage 5 (41.1%). There was no significant difference in the proportion of DM and autoimmune disease from tertile1 to tertile 3. Tertile 3 had less proportion of using statins than tertile 1 (31.9% *vs*. 36.6%, p-trend = 0.03). Stepwise increases in age, history of cardiovascular disease, MAP, BMI, urine PCR, serum WBC count, glycated hemoglobin, ferritin, phosphorus and uric acid levels and stepwise decreases in eGFR, serum hemoglobin, albumin, cholesterol, bicarbonate levels corresponded to the advancement from tertile 1 to tertile 3. In multivariable linear regression analysis, WBC count (β coefficient: 0.08, P<0.001) and ferritin (β coefficient: 0.16, P<0.001) were positively correlated with hsCRP.

**Table 1:** The demographics and clinical characteristics of study cohort.

	High sensitivity c-reactive protein^a^				
	Entire Cohort	Tertile 1	Tertile 2	Tertile 3	P for trend
	N = 3303	N = 1119	N = 1070	N = 1114	
Age (y)	63.5 (13.5)	62.4 (13.2)	63.3 (14.1)	64.8 (13.2)	<0.001
Gender (male) (n[%])	1395 (42.2)	503 (45.0)	443 (41.4)	449 (40.3)	0.03
CKD stage 3 (n[%])	1183 (35.8)	452 (40.4)	404 (37.8)	327 (29.4)	<0.001
CKD stage 4 (n[%])	961 (29.1)	313 (28.0)	319 (29.8)	329 (29.5)	
CKD stage 5 (n[%])	1159 (35.1)	354 (31.6)	347 (32.4)	458 (41.1)	
Diabetes mellitus (n[%])	1472 (44.6)	502 (44.9)	438 (40.9)	532 (47.8)	0.2
Cardiovascular disease (n[%])	873 (26.4)	260 (23.2)	266 (24.9)	347 (31.1)	<0.001
Autoimmune disease (n[%])	105(3.2)	45(4.0)	31(2.9)	29(2.6)	0.1
Cause of chronic kidney disease					0.2
Glomerulonephritis (n[%])	1168 (35.4)	397 (35.5)	396 (37.0)	375 (33.7)	
Tubulointerstitial nephritis (n[%])	300 (9.1)	105 (9.4)	107 (10.0)	88 (7.9)	
Diabetes Mellitus (n[%])	1258 (38.1)	434 (38.8)	366 (34.2)	458 (41.2)	
Hypertension (n[%])	368 (11.1)	125 (11.2)	129 (12.1)	114 (10.2)	
Current smoking status (n[%])	367 (11.1)	109 (9.7)	121 (11.3)	137 (12.3)	0.1
Statins use (n[%])	1081 (32.7)	406 (36.3)	320 (29.9)	355 (31.9)	0.03
Mean arterial pressure (mmHg)	100.0 (13.8)	99.6 (13.3)	99.4 (13.7)	101.0 (14.3)	0.02
Body mass index	24.7 (4.0)	24.3 (3.9)	24.8 (3.8)	25.0 (4.1)	<0.001
High sensitivity c-reactive protein (mg/L)	1.2 (0.4,5.4)	0.2 (0.1,0.4)	1.2 (0.8,1.9)	10.2 (5.3,22.1)	<0.001
eGFR (mL/min/1.73 m^2^)	24.7 (15.1)	26.3 (15.1)	25.6 (15.5)	22.2 (14.3)	<0.001
Hemoglobin (g/dL)	10.9 (2.4)	11.1 (2.4)	11.1 (2.4)	10.6 (2.3)	<0.001
White Blood Cell (x10^3^/µL)	7.2 (2.3)	6.7 (1.9)	7.2 (2.2)	7.7 (2.5)	<0.001
Albumin (g/dL)	3.8 (0.5)	3.9 (0.5)	3.9 (0.5)	3.7 (0.5)	<0.001
Total cholesterol (mg/dL)	191 (162,221)	192 (165, 224)	192 (162, 222)	187 (157, 217)	0.001
Triglyceride (mg/dL)	127 (91,184)	123 (86,180)	128 (93,184)	128 (93,186)	0.02
Glycated hemoglobin (%)	6.5 (1.6)	6.4 (1.5)	6.4 (1.6)	6.6 (1.7)	0.01
Ferritin (ng/mL)	200 (107,349)	180 (96,318)	185 (105,331)	234 (126,417)	<0.001
Urine protein-to-creatinine ratio	1.1 (0.4,2.5)	1.0 (0.3,2.4)	1.0 (0.4,2.1)	1.4 (0.5,3.0)	<0.001
Phosphorus (mg/dL)	4.4 (1.3)	4.4 (1.2)	4.4 (1.2)	4.6 (1.4)	<0.001
Bicarbonate (mEq/L)	21.7 (4.4)	22.3 (4.2)	21.8 (4.4)	21.0 (4.5)	<0.001
Uric acid (mg/dL)	7.9 (2.0)	7.8 (1.8)	7.8 (2.0)	8.1 (2.1)	<0.001

*Notes:* Data are expressed as number (percentage) for categorical variables and mean±SD or median (25^th^, 75^th^ percentile) for continuous variables, as appropriate.

Conversion factors for units: eGFR in mL/min/1.73 m^2^ to mL/s/1.73 m^2^, ×0.01667; hemoglobin in g/dL to g/L, ×10; albumin in g/dL to g/L, ×10; calcium in mg/dL to mmol/L, ×0.2495; phosphate in mg/dL to mmol/L, ×0.3229; cholesterol in mg/dL to mmol/L, ×0.02586; triglyceride in mg/dL to mmol/L, ×0.01129; uric acid in mg/dL toµmol/L, ×59.48.

Abbreviations: eGFR, estimated glomerular filtration rate; hsCRP, high-sensitivity C-reactive protein.

a High sensitivity C-reactive protein tertile cut at 0.5, 3 mg/L.

### hsCRP, WBC Count, Ferritin and RRT

Over a mean follow-up period of 3.2±1.6 years, 1080 patients progressed to commencing RRT (RRT rate: 103.8/1000 patient-years). The subjects of the highest tertile of hsCRP were more likely to need RRT than those of the lowest tertile of hsCRP (38% *vs.* 30%, p for trend <0.001), as shown in Table 2. There was a stepwise increase in the proportion of RRT from tertile 1 of WBC count and ferritin to tertile 3 of those (30.9% *v.s.* 34.3%, p-trend = 0.01; 28.7% *v.s.* 38.8%, p- trend <0.001, respectively).

**Table 2:** Renal event and eGFR decline of hs-CRP tertiles^a^.

	Entire cohort (n = 3303)		Tertile 1 (n = 1119)		Tertile 2 (n = 1070)		Tertile 3 (n = 1114)		P for trend
**Follow-up period (year)**	3.0(1. 8,4.7)		3.2(2.0,4.8)		2.9(1.8,4.5)		2.9(1.6,4.7)		<0.001
**eGFR decline (mL/min/1.73** **m^2^/year)**	−2.2(−5.6, −0.1)		−1.9(−4.9,0.0)		−2.2(−5.2, −0.1)		−2.7(−6.6, −0.2)		<0.001
**Renal Event**	Event n(%)	Event rate^b^	Event n(%)	Event rate^b^	Event n(%)	Event rate^b^	Event n(%)	Event rate^b^	
**Dialysis**	1080 (32.7)	103.8	336 (30.0)	90.8	321 (30.0)	97.6	423(38.0)	123.9	<0.001
**Hemodialysis**	957 (29.0)	91.9	292(26.1)	78.8	278(26.0)	84.5	387(34.7)	113	
**Peritoneal dialysis**	116 (3.5)	11.1	43 (3.8)	11.6	41 (3.8)	12.5	32 (3.5)	9.4	
**Transplantation**	7(0.2)	0.7	1(0.1)	0.3	2(0.2)	0.6	4(0.4)	1.2	

*Notes:* Data are expressed as number (percentage) for categorical variables and median (25^th^, 75^th^ percentile) for continuous variables, as appropriate. Conversion factors for units: eGFR in mL/min/1.73 m^2^ to mL/s/1.73 m^2^, ×0.01667.

a High sensitivity C-reactive protein tertile cut at 0.5, 3 mg/L.

b per 1000-person years.

Table 3 presents the longitudinal associations between RRT and stepwise increases in serum hsCRP, WBC count and ferritin levels. For hsCRP analysis, there was a significant association between a stepwise increase in hsCRP level and commencing RRT in unadjusted model of cox regression analysis. However, a stepwise increase in hsCRP level did not significantly correspond to risk for RRT after multivariate adjustment and the increased risk for RRT was observed only in the highest tertile. The adjusted risks for RRT increased 17% (Hazard ratio〔HR〕: 1.17, 95% Confidence interval 〔CI〕: 1.01–1.36) for subjects of tertile 3 compared with those of tertile 1. Besides, after stratified by albumin, the adjusted HR for the highest tertile versus the lowest tertile was 1.19 (95% CI: 1.02–1.38, Table 4).

**Table 3:** Cox proportional hazards regression analysis of risks for renal replacement treatment among different inflammation markers groups.

	Unadjusted	Model 1	Model 2	Model 3	Model 4
	HR(95% CI)	HR(95% CI)	HR(95% CI)	HR(95% CI)	HR(95% CI)
***High sensitivity c-reactive protein (mg/l)*** a					
Tertile 1	1(Reference)	1(Reference)	1(Reference)	1(Reference)	1(Reference)
Tertile 2	1.11 (0.95–1.29)	0.98 (0.84–1.14)	0.97 (0.83–1.13)	1.03 (0.88–1.20)	1.03 (0.88–1.20)
Tertile 3	1.60 (1.39–1.85)	1.17 (1.02–1.36)	1.16 (1.01–1.34)	1.18 (1.01–1.37)	1.17 (1.01–1.36)
P-trend	<0.001	0.02	0.03	0.06	0.07
***White blood cell (×10^3^/µl)*** b					
Tertile 1	1(Reference)	1(Reference)	1(Reference)	1(Reference)	1(Reference)
Tertile 2	1.08 (0.93–1.25)	1.02 (0.88–1.18)	0.98 (0.85–1.14)	1.03 (0.89–1.20)	1.04 (0.89–1.20)
Tertile 3	1.19 (1.03–1.38)	1.08 (0.93–1.26)	1.02 (0.88–1.19)	1.03 (0.88–1.20)	1.01 (0.87–1.18)
P-trend	0.06	0.53	0.86	0.89	0.90
***Ferritin (ng/ml)*** c					
Tertile 1	1(Reference)	1(Reference)	1(Reference)	1(Reference)	1(Reference)
Tertile 2	1.11 (0.95–1.30)	1.17 (1.00–1.36)	1.17 (1.00–1.36)	1.16 (0.99–1.35)	1.16 (0.99–1.35)
Tertile 3	1.70 (1.47–1.97)	1.30 (1.12–1.51)	1.32 (1.14–1.53)	1.21 (1.04–1.41)	1.20 (1.03–1.40)
P-trend	<0.001	0.002	0.001	0.04	0.05

Abbreviations: HR, Hazard Ratio; CI, Confidence Interval.

a High sensitivity C-reactive protein tertile cut at 0.5, 3 mg/L.

b White blood cell tertile cut at 6, 7.7×10^3^/µL.

c Ferritin tertile cut at 132, 288 ng/mL.

Model 1 adjust for age, gender, eGFR, log urine protein-creatinine ratio.

Model 2 adjust for covariates in model 1 plus glycated hemoglobin, mean arterial pressure, diabetes mellitus, cardiovascular disease, current smoking status, statin use.

Model 3 adjust for covariates in model 2 plus serum hemoglobin, albumin, log cholesterol, phosphorus levels, body mass index.

Model 4 adjust for covariates in model 3 plus causes of chronic kidney disease.

**Table 4:** The risks for renal replacement therapy and renal function progression among different inflammation markers groups stratified by serum albumin.

	Renal replacement therapy		Renal function progression	
	Unadjusted	Adjusted^d^	Unadjusted	Adjusted^d^
	HR(95% CI)	HR(95% CI)	OR(95% CI)	OR(95% CI)
***High sensitivity c-reactive protein (mg/l)*** a ***stratified by serum albumin***				
Tertile 1	1(Reference)	1(Reference)	1(Reference)	1(Reference)
Tertile 2	1.14 (0.98–1.32)	1.02 (0.88–1.19)	1.08 (0.89–1.33)	1.15 (0.93–1.44)
Tertile 3	1.25 (1.08–1.45)	1.19 (1.02–1.38)	1.22 (1.00–1.49)	1.40 (1.12–1.76)
P-trend	0.01	0.05	<0.001	0.01
***White blood cell (×10^3^/µl)*** b ***stratified by serum albumin***				
Tertile 1	1(Reference)	1(Reference)	1(Reference)	1(Reference)
Tertile 2	1.05 (0.91–1.22)	1.08 (0.93–1.25)	1.33 (1.09–1.63)	1.19 (0.95–1.50)
Tertile 3	0.90 (0.78–1.05)	0.96 (0.82–1.12)	1.33 (1.08–1.62)	1.12 (0.89–1.41)
P-trend	0.11	0.27	<0.001	0.30
***Ferritin (ng/ml*** ** ***)*** c ***stratified by serum albumin***				
Tertile 1	1(Reference)	1(Reference)	1(Reference)	1(Reference)
Tertile 2	0.99 (0.85–1.16)	1.13 (0.97–1.32)	1.20 (0.98–1.47)	1.29 (1.03–1.62)
Tertile 3	1.38 (1.20–1.60)	1.26 (1.08–1.46)	1.29 (1.06–1.57)	1.34 (1.07–1.68)
P-trend	<0.001	0.01	<0.001	0.03

Abbreviations: HR, Hazard Ratio; OR, Odds Ratio; CI, Confidence Interval.

a High sensitivity C-reactive protein tertile cut at 0.5, 3 mg/L.

b White blood cell tertile cut at 6, 7.7×10^3^/µL.

c Ferritin tertile cut at 132, 288 ng/mL.

d adjusted for age, gender, eGFR, log urine protein-creatinine ratio, glycated hemoglobin, mean arterial pressure, diabetes mellitus, cardiovascular disease, current smoking status, statin use, serum hemoglobin, albumin, log cholesterol, phosphorus levels, body mass index, causes of chronic kidney disease.

For ferritin analysis, the unadjusted HR for RRT and multivariate adjusted HR for RRT in the highest tertile versus the lowest tertile were 1.70 (95% CI: 1.47–1.97) and 1.20 (95% CI: 1.03–1.40), respectively (Table 3). After stratified by albumin, the adjusted HR for the highest tertile versus the lowest tertile was 1.26 (95% CI: 1.08–1.46). There was a significantly positive correlation between a stepwise increase in ferritin level and RRT in adjusted model (p-trend = 0.01, Table 4). However, there was no significant association between increased WBC count and RRT in unadjusted and multivariate analysis.

### hsCRP, WBC Count, Ferritin and Rapid Renal Progression

There were 841 subjects (25.5%) who had rapid renal progression in our cohort. Decline in renal function in the highest tertile of hsCRP group was also significantly faster than in the lowest tertile of hsCRP group (eGFR slope: −2.7(−6.6, −0.2) *v.s.* −1.9(−4.9, 0.0) ml/min/1.73 m^2^/year, P for trend <0.001, Table 2). In Table 5, the significant association between rapid renal progression and hsCRP, WBC count, and ferritin levels was observed in unadjusted logistic regression analysis. The adjusted odds ratio (OR) for the highest tertile versus the lowest tertile in hsCRP and ferritin analysis were 1.40 (95% CI: 1.13–1.77) and 1.32 (95% CI: 1.06–1.67), respectively. After stratified by albumin, the adjusted OR for the highest tertile versus the lowest tertile in hsCRP and ferritin analysis were 1.40 (95% CI: 1.12–1.76) and 1.34 (95% CI: 1.07–1.68), respectively (Table 4). The positive association between rapid renal progression and stepwise increases in hsCRP and ferritin levels was present after multivariate adjustment (p-trend = 0.01 and 0.03, respectively). However, there was no association between WBC count and rapid renal progression in adjusted model.

**Table 5:** Logistic regression analysis of risks for rapid progression of renal function among different inflammation markers groups.

	Unadjusted	Model 1	Model 2	Model 3	Model 4
	OR(95% CI)	OR(95% CI)	OR(95% CI)	OR(95% CI)	OR(95% CI)
***High sensitivity c-reactive protein (mg/l)*** a					
Tertile 1	1(Reference)	1(Reference)	1(Reference)	1(Reference)	1(Reference)
Tertile 2	1.08 (0.87–1.32)	1.11 (0.89–1.39)	1.11 (0.89–1.39)	1.13 (0.90–1.43)	1.15 (0.91–1.45)
Tertile 3	1.61 (1.33–1.96)	1.49 (1.20–1.84)	1.44 (1.18–1.82)	1.38 (1.12–1.75)	1.40 (1.13–1.77)
P-trend	<0.001	0.001	0.001	0.01	0.01
***White blood cell (×10^3^/µl)*** b					
Tertile 1	1(Reference)	1(Reference)	1(Reference)	1(Reference)	1(Reference)
Tertile 2	1.42 (1.15–1.75)	1.30 (1.04–1.63)	1.23 (0.98–1.58)	1.33 (1.07–1.69)	1.32 (1.05–1.67)
Tertile 3	1.89 (1.55–2.32)	1.37 (1.10–1.70)	1.24 (1.01–1.58)	1.23 (0.98–1.56)	1.21 (0.96–1.53)
P-trend	<0.001	0.01	0.07	0.04	0.05
***Ferritin (ng/ml)*** c					
Tertile 1	1(Reference)	1(Reference)	1(Reference)	1(Reference)	1(Reference)
Tertile 2	1.34 (1.09–1.64)	1.33 (1.06–1.66)	1.34 (1.08–1.69)	1.35 (1.08–1.71)	1.34 (1.07–1.69)
Tertile 3	1.67 (1.37–2.05)	1.44 (1.16–1.80)	1.47 (1.19–1.85)	1.34 (1.07–1.69)	1.32 (1.06–1.67)
P-trend	<0.001	0.004	0.002	0.01	0.02

Abbreviations: OR, Odds Ratio; CI, Confidence Interval.

a High sensitivity C-reactive protein tertile cut at 0.5, 3 mg/L.

b White blood cell tertile cut at 6, 7.7×10^3^/µL.

c Ferritin tertile cut at 132, 288 ng/mL.

Model 1 adjust for age, gender, eGFR, log urine protein-creatinine ratio.

Model 2 adjust for covariates in model 1 plus glycated hemoglobin, mean arterial pressure, diabetes mellitus, cardiovascular disease, current smoking status, statin use.

Model 3 adjust for covariates in model 2 plus serum hemoglobin, albumin, log cholesterol, phosphorus levels, body mass index.

Model 4 adjust for covariates in model 3 plus causes of chronic kidney disease.

### Sensitivity Analysis

For the first sensitivity analysis, hsCRP was divided into 4 groups, cut by 0.5, 3, and 10 mg/L. The adjusted HR of RRT for the subjects with the highest level of hsCRP versus those with the lowest level of hsCRP was 1.19 (95% CI: 1.00–1.42, table S1). For the second sensitivity analysis, hsCRP was also divided into 3 groups, cut by 1 and 3 mg/L. hsCRP level above 3 mg/L was associated with increased risk for RRT in the adjusted model (HR:1.15, 95% CI:1.00–1.32, table S2). The results of this cox regression analysis were consistent with the original analyses.

## Discussion

A number of studies have documented a significant association between inflammation and cardiovascular disease and mortality, and inflammation has been considered as an independent of predictor of adverse outcomes [7], [8]. Inflammation itself is a principal component of histological features of almost all kidneys diseases [2]. However, the relationship between inflammation and progression of renal function impairment in CKD patients has not been well addressed. This study is the first to show that high levels of inflammation markers, hsCRP and ferritin, are associated with commencing RRT and rapid renal progression in patients with stages 3–5 CKD. Patients with the highest tertile of hsCRP and ferritin have a 1.2-fold risk for RRT and 1.4-fold risk for rapid renal progression.

One of our novel findings is that hsCRP is an independent risk factor for RRT and rapid renal function decline. Traditionally, hsCRP has been regarded as a predictor of future risk for heart attack, stroke, sudden cardiac death, and the development of peripheral arterial disease [24]. AHA demonstrates that levels of CRP less than 1, 1 to 3, and greater than 3 mg/L discriminate between individuals with low, moderate, and high risk for future cardiovascular event. Based on our results, patients with hsCRP level above 3 mg/L are more likely to enter maintenance dialysis and the status of rapid renal function progression. For CKD patients, the level of CRP in excess of 3 mg/L suggests quite elevated renal risk. In addition, in our subgroup analysis, higher levels of hsCRP were associated with progression to RRT in subjects with eGFR<30 ml/min/1.73 m^2^, not in those with eGFR ≧30 ml/min/1.73 m^2^ (p-interaction = 0.02, data not shown). hsCRP could be even more predictive in patients with later stages than those in earlier stages and this was consistent with the observation of Shankar et al [19]. Therefore, for late CKD cohort with hsCRP level above 3 mg/L, clinicians need to pay more attention to their renal function decline.

Our study result suggests that elevated levels of ferritin are associated with rapid renal function decline and progression to RRT in stages 3–5 CKD patients. Serum ferritin not only reflects iron store but is also an acute phase reactant. In our cohort, only 5% of the patients at time of enrollment and up to 12% of the patients during the observation period received iron supplementation. Inflammatory cytokines would increase the synthesis of both H and L subunits of ferritin [25], [26]. Ferritin elicits macrophage accumulation and augments ROS formation during inflammation [12]. Kalantar-Zadeh et.al showed a significant association between ferritin and malnutrition-inflammation complex syndrome, which plays a central role in poor clinical outcome in dialysis patients [13]. Ferritin had been considered as an independent risk factor for mortality and hospitalization in dialysis patients [27]. However, the relationship between ferritin and renal function decline in CKD patients had been not evaluated in the past literature. As a trend for higher mortality in late stages of CKD patients with serum ferritin level above 250 ng/ml [28], our results suggest that CKD patients with ferritin level above 288 ng/ml are more likely to receive adverse renal outcome.

On the other hand, WBC count has been a general indicator of inflammation and infection, and was significantly associated with development of CKD and renal function change in the general population [16]–[19]. However, as the results of Kovesdy et.al’s study demonstrate [20], WBC count did not suffice to be a positive predictor of RRT and rapid renal progression in our cohort. The reasons causing inconclusive results between studies are not clear. A major difference is that the effect of baseline renal function, such as eGFR and urine protein-creatinine ratio, was not taken into consideration in previous studies. Besides, some reports suggested that lower lymphocyte count was associated with malnutrition and inflammation in dialysis patients [29], [30]. The lower percentage of lymphocytes in WBC count was also correlated with increased risk for all-cause mortality and initiation of maintenance dialysis [20]. Because of comprising many classifications, such as leukocyte and lymphocyte, WBC might not be specific enough to be a predictor. Therefore, instead of WBC count, lymphocyte count seems to be a more precise predictor of clinical outcome in CKD cohort.

The potential mechanisms responsible for the association of inflammation with renal function decline are not clear. In vitro experiments showed that CRP reduced the release of basal and stimulated nitric oxide and might lead to oxidative stress which might be related to renal function decline [31]. In addition, high circulating CRP levels might induce injuries through deposition in the glomerular endothelium and reduce functional renal mass, and contribute to renal scaring, interstitial fibrosis, and tubular hypertrophy through several hypothesized mechanism, such as increased angiotensin II levels and elevated transforming growth factor-β levels [32], [33]. From the aspect of a clinical view, inflammation and malnutrition are usually considered together because inflammation increases catabolism and decreases synthesis of proteins, leading to protein energy malnutrition (PEM) [34]. PEM may lead to oxidative stress, which can increase activity of pro-inflammatory cytokines [35]. PEM may decrease host defense and predispose patients to latent or overt infection and inflammation [36]. Some studies opined that both inflammation and malnutrition have been recognized as important risk factors of poor clinical outcome in patients on dialysis [37], [38]. However, some studies indicated that albumin, as a marker of malnutrition, and not CRP, was associated with mortality in dialysis patients [39]. The results of previous reports are inconsistent in dialysis cohort and a consensus about the association between inflammation and malnutrition has not been reached in CKD cohort. The present study used stratification of albumin to analyze the influence of the interaction between inflammation and albumin on renal outcome and the results revealed inflammation as an independent predictor of renal outcome. It means that the interaction between inflammation and malnutrition may not be so strong in CKD patients compared to dialysis patients. In CKD patients, malnutrition may only play a partial role in an inflammation process. Besides, the association between CKD progression and inflammation may be confounded by several co-morbidities, (such as obesity, DM, dyslipidemia, hypertension and CVD), which were traditional risk factors for renal function decline [40]–[43]. Inflammation may mediate most of the effects of traditional risk factors on the kidney and facilitate renal function deterioration. A recent study also reported that Bardoxolone methyl, an oral antioxidant inflammation modulator was associated with improvement in the estimated GFR in patients with advanced CKD [44]. Because of this complicated interaction among traditional risk factors, inflammation and CKD, we adjusted these factors in multivariate analysis to minimize their effects. Besides, we also performed subgroup analyses in non-DM and non-CVD patients to eliminate the influences of co-morbidities on renal outcome and our findings revealed that inflammation was still significantly correlated with poor renal outcome (data not shown). Therefore, inflammation itself is possibly an independent mediator of renal function progression in CKD cohort.

This study has some limitations that must be considered. First, the effect of CRP variation over time has to be considered. Several studies showed that a single CRP value could predict cardiovascular events and mortality in dialysis patients [45]–[47]. However, hsCRP might vary during the follow-up period and it’s likely that the time point chosen for study would jeopardize the reliability. Ates et al. indicated that averaged level of CRP during follow-up is more predictive of prognosis compared to the baseline CRP level in dialysis patients [48]. Therefore, we used the time-averaged level of hsCRP, three months before and after the enrollment, to avoid the potential interference from CRP variability. Second, the study cohort is a patient collection by referral and they may be already at higher risk for progression, and the extrapolated utilization in different CKD cohorts will be limited.

In conclusion, our study demonstrates that, during a 3-year follow-up period, high levels of hsCRP and ferritin were associated with increased risks for RRT and rapid renal progression in stages 3–5 CKD patients. Future studies will be necessary to investigate the pathogenic link between inflammation and renal function progression in order to salvage the failing kidneys through this common factor in almost all kidney diseases.

## Supporting Information

Table S1 **Sensitivity analysis - Cox proportional hazards regression analysis of risks for renal replacement treatment according to different divisions of hsCRP.**
(DOC)

Table S2 **Sensitivity analysis - Cox proportional hazards regression analysis of risks for renal replacement treatment according to different divisions of hsCRP.**
(DOC)
