# Supplementary material for: Association of hsCRP, White Blood Cell Count and Ferritin with Renal Outcome in Chronic Kidney Disease Patients
Source: PLoS One. 2012 Dec 31;7(12):e52775. doi: 10.1371/journal.pone.0052775 (PMC3534111; doi:10.1371/journal.pone.0052775)
Supplement: Table S2 — Sensitivity analysis - Cox proportional hazards regression analysis of risks for renal replacement treatment according to different divisions of hsCRP. (DOC) [file pone.0052775.s002.doc]

Table S2. Sensitivity analysis - Cox proportional hazards regression analysis of risks for renal replacement treatment according to different divisions of hsCRP

| HR(95% CI) | hsCRP≦1mg/L | 1mg/L<hsCRP≦3mg/L | hsCRP>3mg/L | P- trend |
| --- | --- | --- | --- | --- |
| Unadjusted | 1 (reference) | 1.07 (0.90-1.27) | 1.55 (1.36-1.77) | <0.001 |
| Model 1 | 1 (reference) | 1.04 (0.87-1.23) | 1.20 (1.05-1.37) | 0.03 |
| Model 2 | 1 (reference) | 1.01 (0.85-1.20) | 1.18 (1.03-1.35) | 0.03 |
| Model 3 | 1 (reference) | 0.98 (0.83-1.17) | 1.16 (1.01-1.32) | 0.13 |
| Model 4 | 1 (reference) | 0.98 (0.83-1.17) | 1.15 (1.00-1.32) | 0.15 |

Model 1 adjust for age, gender, eGFR, log urine protein-creatinine ratio

Model 2 adjust for covariates in model 1 plus glycated hemoglobin, mean arterial pressure, diabetes mellitus, cardiovascular disease, current smoking status, statin.

Model 3 adjust for covariates in model 2 plus serum hemoglobin, albumin, log cholesterol, phosphorus levels, body mass index

Model 4 adjust for covariates in model 3 plus causes of chronic kidney disease
